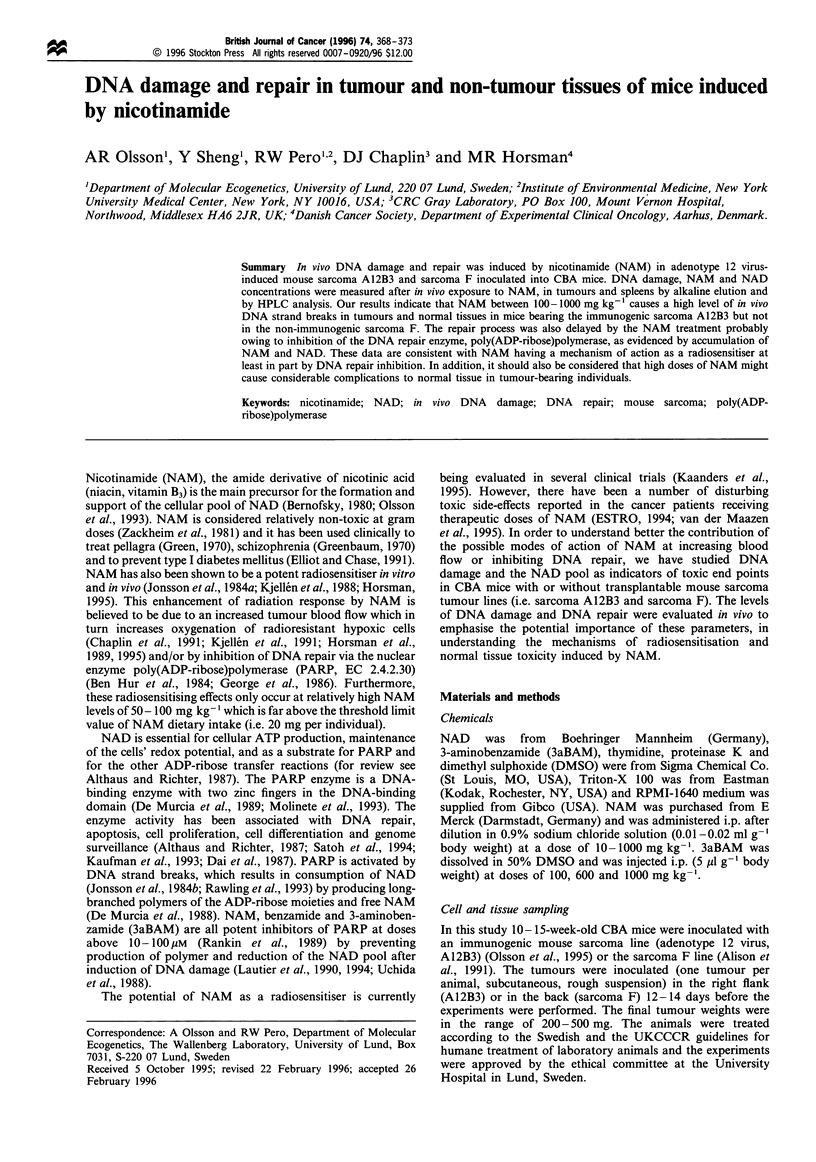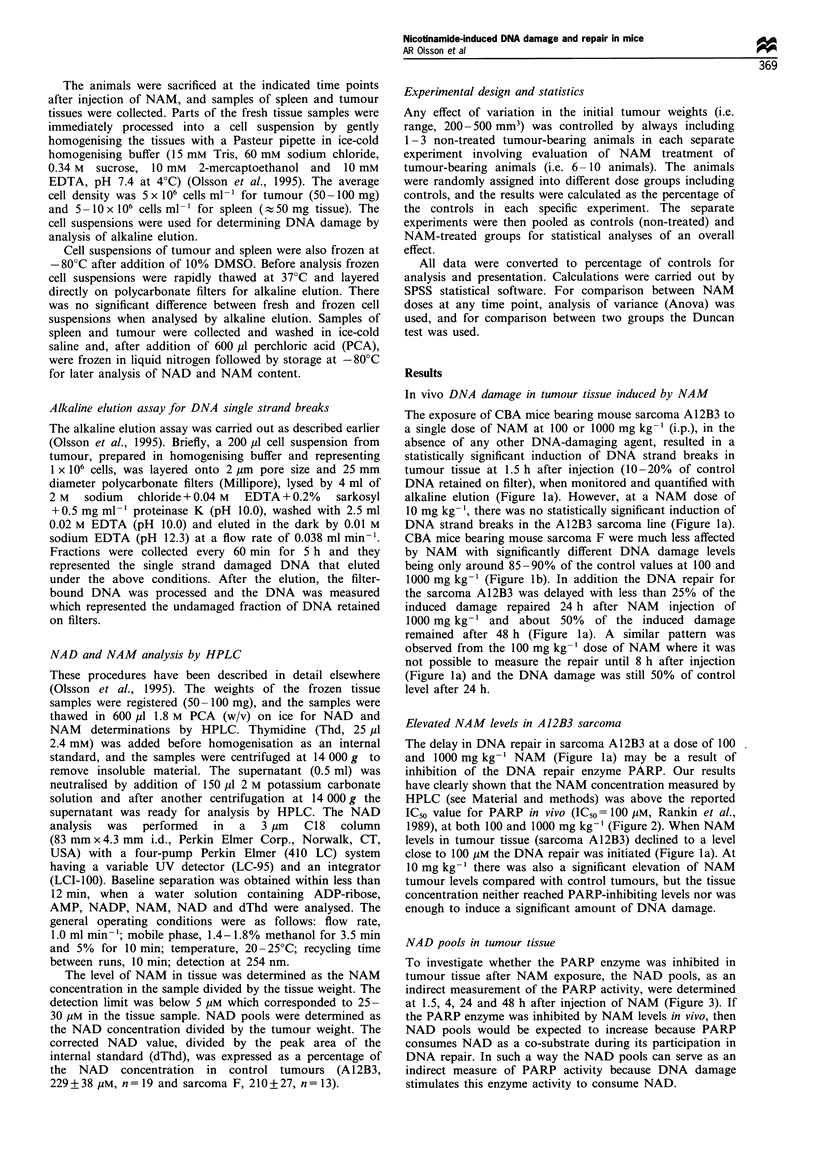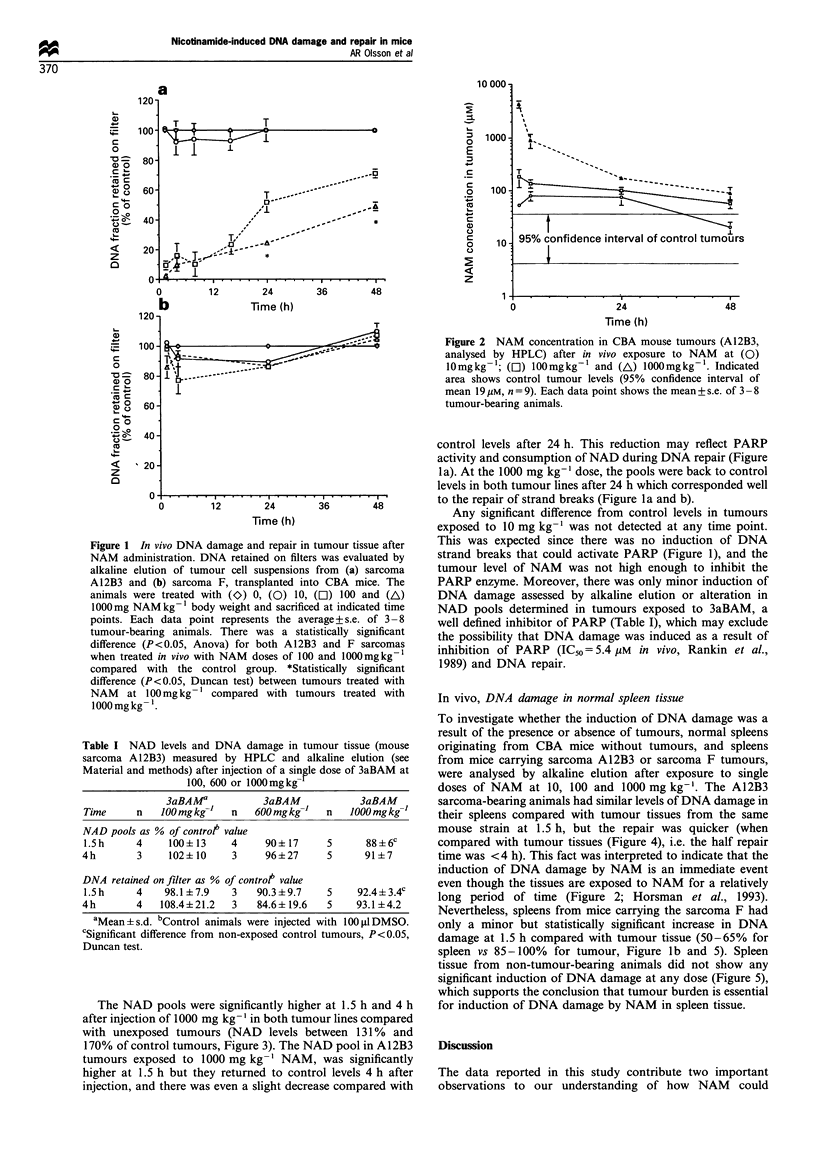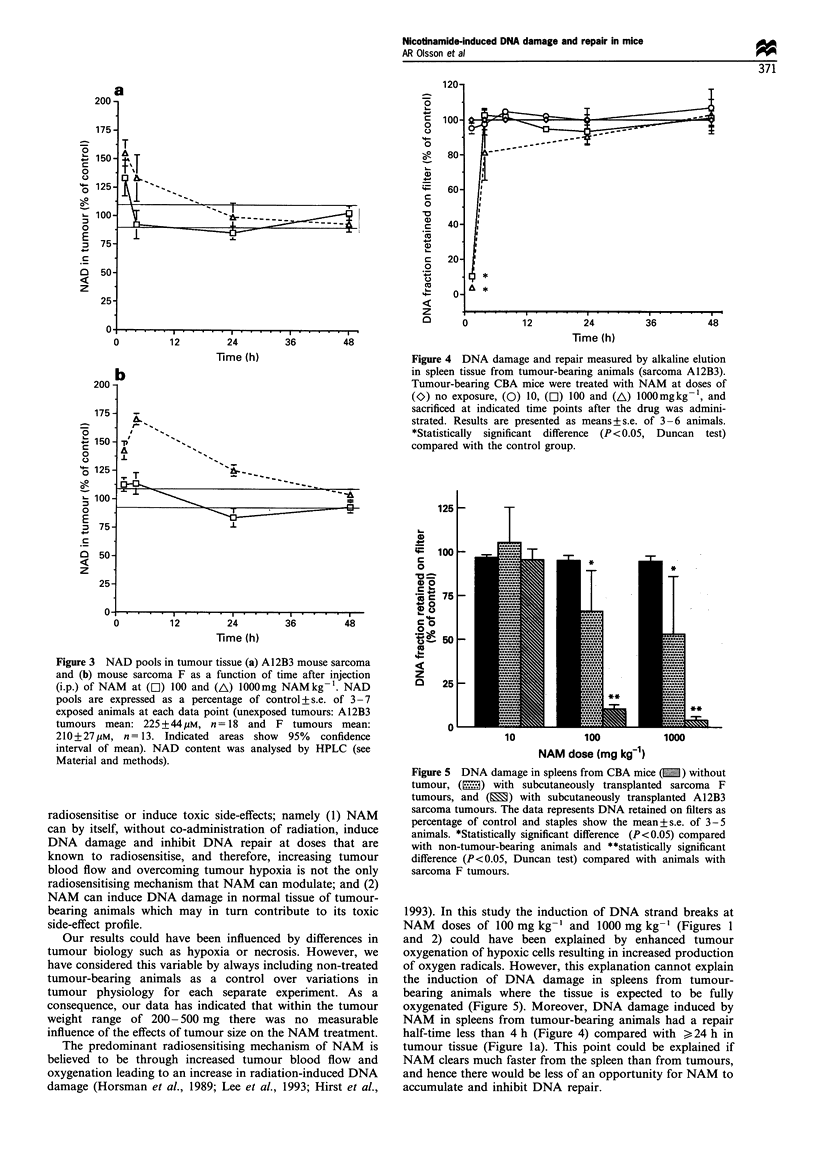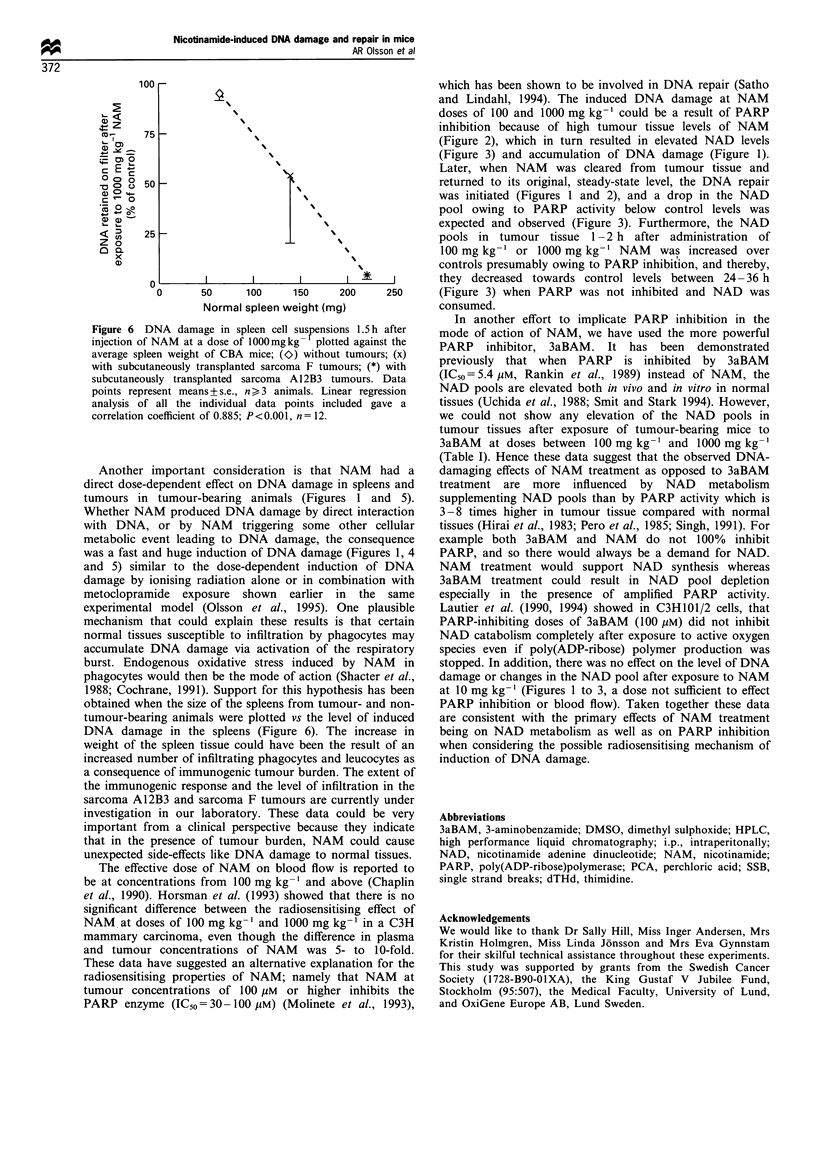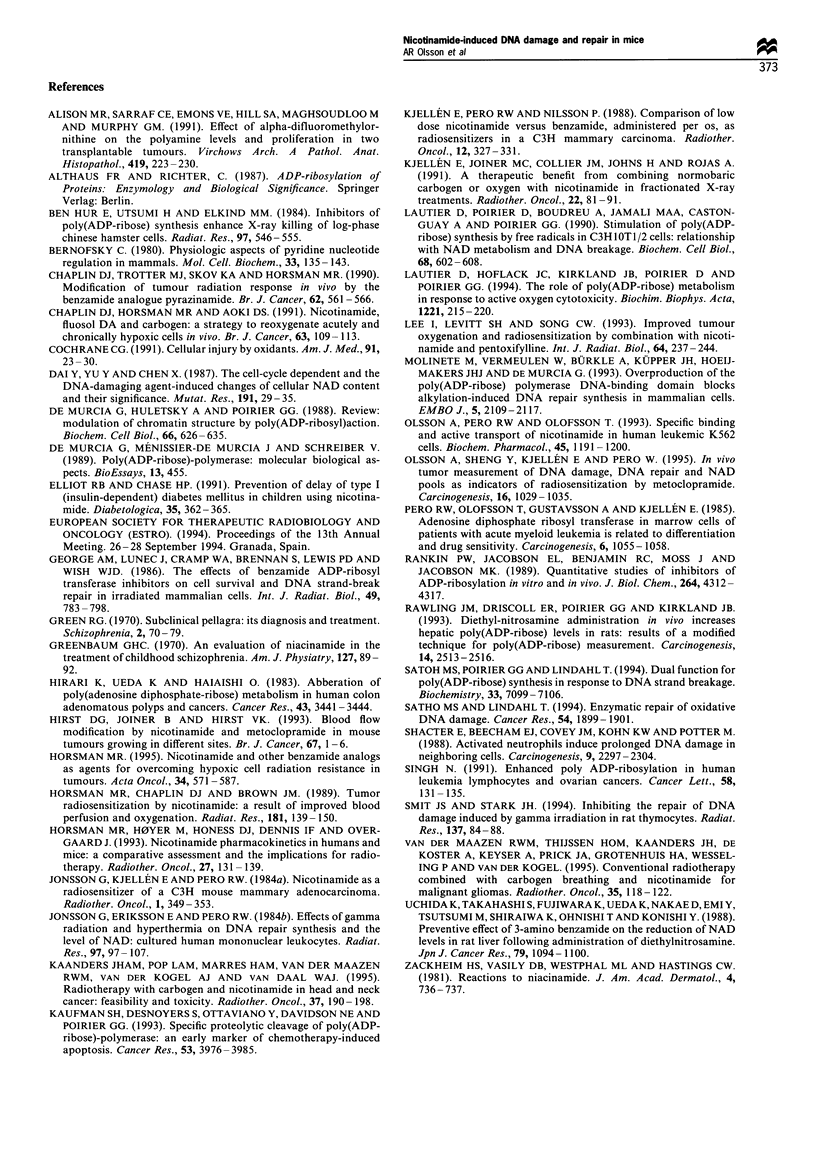# DNA damage and repair in tumour and non-tumour tissues of mice induced by nicotinamide.

**DOI:** 10.1038/bjc.1996.367

**Published:** 1996-08

**Authors:** A. R. Olsson, Y. Sheng, R. W. Pero, D. J. Chaplin, M. R. Horsman

**Affiliations:** Department of Molecular Ecogenetics, University of Lund, Sweden.

## Abstract

In vivo DNA damage and repair was induced by nicotinamide (NAM) in adenotype 12 virus-induced mouse sarcoma A12B3 and sarcoma F inoculated into CBA mice. DNA damage, NAM and NAD concentrations were measured after in vivo exposure to NAM, in tumours and spleens by alkaline elution and by HPLC analysis. Our results indicate that NAM between 100-1000 mg kg-1 causes a high level of in vivo DNA strand breaks in tumours and normal tissues in mice bearing the immunogenic sarcoma A12B3 but not in the non-immunogenic sarcoma F. The repair process was also delayed by the NAM treatment probably owing to inhibition of the DNA repair enzyme, poly(ADP-ribose)polymerase, as evidenced by accumulation of NAM and NAD. These data are consistent with NAM having a mechanism of action as a radiosensitiser at least in part by DNA repair inhibition. In addition, it should also be considered that high doses of NAM might cause considerable complications to normal tissue in tumour-bearing individuals.


British Journal of Cancer (1996) 74, 368-373
© 1996 Stockton Press All rights reserved 0007-0920/96 $12.00

### DNA damage and repair in tumour and non-tumour tissues of mice induced by nicotinamide

AR Olsson[1], Y Sheng[1], RW Pero[1,2], DJ Chaplin[3] and MR Horsman[4]

[1]Department of Molecular Ecogenetics, University of Lund, 220 07 Lund, Sweden; [2]Institute of Environmental Medicine, New York University Medical Center, New York, NY 10016, USA; [3]CRC Gray Laboratory, PO Box 100, Mount Vernon Hospital, Northwood, Middlesex HA6 2JR, UK; [4]Danish Cancer Society, Department of Experimental Clinical Oncology, Aarhus, Denmark.

**Summary** *In vivo* DNA damage and repair was induced by nicotinamide (NAM) in adenotype 12 virus-induced mouse sarcoma A12B3 and sarcoma F inoculated into CBA mice. DNA damage, NAM and NAD concentrations were measured after *in vivo* exposure to NAM, in tumours and spleens by alkaline elution and by HPLC analysis. Our results indicate that NAM between 100-1000 mg $kg^{-1}$ causes a high level of *in vivo* DNA strand breaks in tumours and normal tissues in mice bearing the immunogenic sarcoma A12B3 but not in the non-immunogenic sarcoma F. The repair process was also delayed by the NAM treatment probably owing to inhibition of the DNA repair enzyme, poly(ADP-ribose)polymerase, as evidenced by accumulation of NAM and NAD. These data are consistent with NAM having a mechanism of action as a radiosensitiser at least in part by DNA repair inhibition. In addition, it should also be considered that high doses of NAM might cause considerable complications to normal tissue in tumour-bearing individuals.

**Keywords:** nicotinamide; NAD; *in vivo* DNA damage; DNA repair; mouse sarcoma; poly(ADP-ribose)polymerase

Nicotinamide (NAM), the amide derivative of nicotinic acid (niacin, vitamin $B_3$) is the main precursor for the formation and support of the cellular pool of NAD (Bernofsky, 1980; Olsson *et al.*, 1993). NAM is considered relatively non-toxic at gram doses (Zackheim *et al.*, 1981) and it has been used clinically to treat pellagra (Green, 1970), schizophrenia (Greenbaum, 1970) and to prevent type I diabetes mellitus (Elliot and Chase, 1991). NAM has also been shown to be a potent radiosensitiser *in vitro* and *in vivo* (Jonsson *et al.*, 1984*a*; Kjellén *et al.*, 1988; Horsman, 1995). This enhancement of radiation response by NAM is believed to be due to an increased tumour blood flow which in turn increases oxygenation of radioresistant hypoxic cells (Chaplin *et al.*, 1991; Kjellén *et al.*, 1991; Horsman *et al.*, 1989, 1995) and/or by inhibition of DNA repair via the nuclear enzyme poly(ADP-ribose)polymerase (PARP, EC 2.4.2.30) (Ben Hur *et al.*, 1984; George *et al.*, 1986). Furthermore, these radiosensitising effects only occur at relatively high NAM levels of 50-100 mg $kg^{-1}$ which is far above the threshold limit value of NAM dietary intake (i.e. 20 mg per individual).

NAD is essential for cellular ATP production, maintenance of the cells' redox potential, and as a substrate for PARP and for the other ADP-ribose transfer reactions (for review see Althaus and Richter, 1987). The PARP enzyme is a DNA-binding enzyme with two zinc fingers in the DNA-binding domain (De Murcia *et al.*, 1989; Molinete *et al.*, 1993). The enzyme activity has been associated with DNA repair, apoptosis, cell proliferation, cell differentiation and genome surveillance (Althaus and Richter, 1987; Satoh *et al.*, 1994; Kaufman *et al.*, 1993; Dai *et al.*, 1987). PARP is activated by DNA strand breaks, which results in consumption of NAD (Jonsson *et al.*, 1984*b*; Rawling *et al.*, 1993) by producing long-branched polymers of the ADP-ribose moieties and free NAM (De Murcia *et al.*, 1988). NAM, benzamide and 3-aminobenzamide (3aBAM) are all potent inhibitors of PARP at doses above 10-100 $\mu$M (Rankin *et al.*, 1989) by preventing production of polymer and reduction of the NAD pool after induction of DNA damage (Lautier *et al.*, 1990, 1994; Uchida *et al.*, 1988).

The potential of NAM as a radiosensitiser is currently being evaluated in several clinical trials (Kaanders *et al.*, 1995). However, there have been a number of disturbing toxic side-effects reported in the cancer patients receiving therapeutic doses of NAM (ESTRO, 1994; van der Maazen *et al.*, 1995). In order to understand better the contribution of the possible modes of action of NAM at increasing blood flow or inhibiting DNA repair, we have studied DNA damage and the NAD pool as indicators of toxic end points in CBA mice with or without transplantable mouse sarcoma tumour lines (i.e. sarcoma A12B3 and sarcoma F). The levels of DNA damage and DNA repair were evaluated *in vivo* to emphasise the potential importance of these parameters, in understanding the mechanisms of radiosensitisation and normal tissue toxicity induced by NAM.

Correspondence: A Olsson and RW Pero, Department of Molecular Ecogenetics, The Wallenberg Laboratory, University of Lund, Box 7031, S-220 07 Lund, Sweden
Received 5 October 1995; revised 22 February 1996; accepted 26 February 1996

### Materials and methods

### Chemicals

NAD was from Boehringer Mannheim (Germany), 3-aminobenzamide (3aBAM), thymidine, proteinase K and dimethyl sulphoxide (DMSO) were from Sigma Chemical Co. (St Louis, MO, USA), Triton-X 100 was from Eastman (Kodak, Rochester, NY, USA) and RPMI-1640 medium was supplied from Gibco (USA). NAM was purchased from E Merck (Darmstadt, Germany) and was administered i.p. after dilution in 0.9% sodium chloride solution (0.01-0.02 ml $g^{-1}$ body weight) at a dose of 10-1000 mg $kg^{-1}$. 3aBAM was dissolved in 50% DMSO and was injected i.p. (5 $\mu$l $g^{-1}$ body weight) at doses of 100, 600 and 1000 mg $kg^{-1}$.

### Cell and tissue sampling

In this study 10-15-week-old CBA mice were inoculated with an immunogenic mouse sarcoma line (adenotype 12 virus, A12B3) (Olsson *et al.*, 1995) or the sarcoma F line (Alison *et al.*, 1991). The tumours were inoculated (one tumour per animal, subcutaneous, rough suspension) in the right flank (A12B3) or in the back (sarcoma F) 12-14 days before the experiments were performed. The final tumour weights were in the range of 200-500 mg. The animals were treated according to the Swedish and the UKCCCR guidelines for humane treatment of laboratory animals and the experiments were approved by the ethical committee at the University Hospital in Lund, Sweden.

The animals were sacrificed at the indicated time points after injection of NAM, and samples of spleen and tumour tissues were collected. Parts of the fresh tissue samples were immediately processed into a cell suspension by gently homogenising the tissues with a Pasteur pipette in ice-cold homogenising buffer (15 mM Tris, 60 mM sodium chloride, 0.34 M sucrose, 10 mM 2-mercaptoethanol and 10 mM EDTA, pH 7.4 at 4°C) (Olsson *et al.*, 1995). The average cell density was $5 \times 10^6$ cells $ml^{-1}$ for tumour (50–100 mg) and $5-10 \times 10^6$ cells $ml^{-1}$ for spleen ($\approx 50$ mg tissue). The cell suspensions were used for determining DNA damage by analysis of alkaline elution.

Cell suspensions of tumour and spleen were also frozen at −80°C after addition of 10% DMSO. Before analysis frozen cell suspensions were rapidly thawed at 37°C and layered directly on polycarbonate filters for alkaline elution. There was no significant difference between fresh and frozen cell suspensions when analysed by alkaline elution. Samples of spleen and tumour were collected and washed in ice-cold saline and, after addition of 600 μl perchloric acid (PCA), were frozen in liquid nitrogen followed by storage at −80°C for later analysis of NAD and NAM content.

*Alkaline elution assay for DNA single strand breaks*

The alkaline elution assay was carried out as described earlier (Olsson *et al.*, 1995). Briefly, a 200 μl cell suspension from tumour, prepared in homogenising buffer and representing $1 \times 10^6$ cells, was layered onto 2 μm pore size and 25 mm diameter polycarbonate filters (Millipore), lysed by 4 ml of 2 M sodium chloride + 0.04 M EDTA + 0.2% sarkosyl + 0.5 mg $ml^{-1}$ proteinase K (pH 10.0), washed with 2.5 ml 0.02 M EDTA (pH 10.0) and eluted in the dark by 0.01 M sodium EDTA (pH 12.3) at a flow rate of 0.038 ml $min^{-1}$. Fractions were collected every 60 min for 5 h and they represented the single strand damaged DNA that eluted under the above conditions. After the elution, the filter-bound DNA was processed and the DNA was measured which represented the undamaged fraction of DNA retained on filters.

*NAD and NAM analysis by HPLC*

These procedures have been described in detail elsewhere (Olsson *et al.*, 1995). The weights of the frozen tissue samples were registered (50–100 mg), and the samples were thawed in 600 μl 1.8 M PCA (w/v) on ice for NAD and NAM determinations by HPLC. Thymidine (Thd, 25 μl 2.4 mM) was added before homogenisation as an internal standard, and the samples were centrifuged at 14 000 *g* to remove insoluble material. The supernatant (0.5 ml) was neutralised by addition of 150 μl 2 M potassium carbonate solution and after another centrifugation at 14 000 *g* the supernatant was ready for analysis by HPLC. The NAD analysis was performed in a 3 μm C18 column (83 mm × 4.3 mm i.d., Perkin Elmer Corp., Norwalk, CT, USA) with a four-pump Perkin Elmer (410 LC) system having a variable UV detector (LC-95) and an integrator (LCI-100). Baseline separation was obtained within less than 12 min, when a water solution containing ADP-ribose, AMP, NADP, NAM, NAD and dThd were analysed. The general operating conditions were as follows: flow rate, 1.0 ml $min^{-1}$; mobile phase, 1.4–1.8% methanol for 3.5 min and 5% for 10 min; temperature, 20–25°C; recycling time between runs, 10 min; detection at 254 nm.

The level of NAM in tissue was determined as the NAM concentration in the sample divided by the tissue weight. The detection limit was below 5 μM which corresponded to 25–30 μM in the tissue sample. NAD pools were determined as the NAD concentration divided by the tumour weight. The corrected NAD value, divided by the peak area of the internal standard (dThd), was expressed as a percentage of the NAD concentration in control tumours (A12B3, $229 \pm 38$ μM, $n = 19$ and sarcoma F, $210 \pm 27$, $n = 13$).

*Experimental design and statistics*

Any effect of variation in the initial tumour weights (i.e. range, 200–500 $mm^3$) was controlled by always including 1–3 non-treated tumour-bearing animals in each separate experiment involving evaluation of NAM treatment of tumour-bearing animals (i.e. 6–10 animals). The animals were randomly assigned into different dose groups including controls, and the results were calculated as the percentage of the controls in each specific experiment. The separate experiments were then pooled as controls (non-treated) and NAM-treated groups for statistical analyses of an overall effect.

All data were converted to percentage of controls for analysis and presentation. Calculations were carried out by SPSS statistical software. For comparison between NAM doses at any time point, analysis of variance (Anova) was used, and for comparison between two groups the Duncan test was used.

### Results

In vivo *DNA damage in tumour tissue induced by NAM*

The exposure of CBA mice bearing mouse sarcoma A12B3 to a single dose of NAM at 100 or 1000 mg $kg^{-1}$ (i.p.), in the absence of any other DNA-damaging agent, resulted in a statistically significant induction of DNA strand breaks in tumour tissue at 1.5 h after injection (10–20% of control DNA retained on filter), when monitored and quantified with alkaline elution (Figure 1a). However, at a NAM dose of 10 mg $kg^{-1}$, there was no statistically significant induction of DNA strand breaks in the A12B3 sarcoma line (Figure 1a). CBA mice bearing mouse sarcoma F were much less affected by NAM with significantly different DNA damage levels being only around 85–90% of the control values at 100 and 1000 mg $kg^{-1}$ (Figure 1b). In addition the DNA repair for the sarcoma A12B3 was delayed with less than 25% of the induced damage repaired 24 h after NAM injection of 1000 mg $kg^{-1}$ and about 50% of the induced damage remained after 48 h (Figure 1a). A similar pattern was observed from the 100 mg $kg^{-1}$ dose of NAM where it was not possible to measure the repair until 8 h after injection (Figure 1a) and the DNA damage was still 50% of control level after 24 h.

*Elevated NAM levels in A12B3 sarcoma*

The delay in DNA repair in sarcoma A12B3 at a dose of 100 and 1000 mg $kg^{-1}$ NAM (Figure 1a) may be a result of inhibition of the DNA repair enzyme PARP. Our results have clearly shown that the NAM concentration measured by HPLC (see Material and methods) was above the reported $IC_{50}$ value for PARP *in vivo* ($IC_{50} = 100$ μM, Rankin *et al.*, 1989), at both 100 and 1000 mg $kg^{-1}$ (Figure 2). When NAM levels in tumour tissue (sarcoma A12B3) declined to a level close to 100 μM the DNA repair was initiated (Figure 1a). At 10 mg $kg^{-1}$ there was also a significant elevation of NAM tumour levels compared with control tumours, but the tissue concentration neither reached PARP-inhibiting levels nor was enough to induce a significant amount of DNA damage.

*NAD pools in tumour tissue*

To investigate whether the PARP enzyme was inhibited in tumour tissue after NAM exposure, the NAD pools, as an indirect measurement of the PARP activity, were determined at 1.5, 4, 24 and 48 h after injection of NAM (Figure 3). If the PARP enzyme was inhibited by NAM levels *in vivo*, then NAD pools would be expected to increase because PARP consumes NAD as a co-substrate during its participation in DNA repair. In such a way the NAD pools can serve as an indirect measure of PARP activity because DNA damage stimulates this enzyme activity to consume NAD.

**Figure 1** *In vivo* DNA damage and repair in tumour tissue after NAM administration. DNA retained on filters was evaluated by alkaline elution of tumour cell suspensions from (**a**) sarcoma A12B3 and (**b**) sarcoma F, transplanted into CBA mice. The animals were treated with (◇) 0, (○) 10, (□) 100 and (△) 1000 mg NAM kg$^{-1}$ body weight and sacrificed at indicated time points. Each data point represents the average±s.e. of 3–8 tumour-bearing animals. There was a statistically significant difference ($P<0.05$, Anova) for both A12B3 and F sarcomas when treated *in vivo* with NAM doses of 100 and 1000 mg kg$^{-1}$ compared with the control group. *Statistically significant difference ($P<0.05$, Duncan test) between tumours treated with NAM at 100 mg kg$^{-1}$ compared with tumours treated with 1000 mg kg$^{-1}$.

**Table I** NAD levels and DNA damage in tumour tissue (mouse sarcoma A12B3) measured by HPLC and alkaline elution (see Material and methods) after injection of a single dose of 3aBAM at 100, 600 or 1000 mg kg$^{-1}$

| *Time* | n | *3aBAM*[a] *100 mg kg*$^{-1}$ | n | *3aBAM 600 mg kg*$^{-1}$ | n | *3aBAM 1000 mg kg*$^{-1}$ |
|---|---|---|---|---|---|---|
| *NAD pools as % of control*[b] *value* | | | | | | |
| 1.5 h | 4 | 100±13 | 4 | 90±17 | 5 | 88±6[c] |
| 4 h | 3 | 102±10 | 3 | 96±27 | 5 | 91±7 |
| *DNA retained on filter as % of control*[b] *value* | | | | | | |
| 1.5 h | 4 | 98.1±7.9 | 3 | 90.3±9.7 | 5 | 92.4±3.4[c] |
| 4 h | 4 | 108.4±21.2 | 3 | 84.6±19.6 | 5 | 93.1±4.2 |

[a]Mean±s.d. [b]Control animals were injected with 100 µl DMSO. [c]Significant difference from non-exposed control tumours, $P<0.05$, Duncan test.

The NAD pools were significantly higher at 1.5 h and 4 h after injection of 1000 mg kg$^{-1}$ in both tumour lines compared with unexposed tumours (NAD levels between 131% and 170% of control tumours, Figure 3). The NAD pool in A12B3 tumours exposed to 1000 mg kg$^{-1}$ NAM, was significantly higher at 1.5 h but they returned to control levels 4 h after injection, and there was even a slight decrease compared with

**Figure 2** NAM concentration in CBA mouse tumours (A12B3, analysed by HPLC) after *in vivo* exposure to NAM at (○) 10 mg kg$^{-1}$; (□) 100 mg kg$^{-1}$ and (△) 1000 mg kg$^{-1}$. Indicated area shows control tumour levels (95% confidence interval of mean 19 µM, $n=9$). Each data point shows the mean±s.e. of 3–8 tumour-bearing animals.

control levels after 24 h. This reduction may reflect PARP activity and consumption of NAD during DNA repair (Figure 1a). At the 1000 mg kg$^{-1}$ dose, the pools were back to control levels in both tumour lines after 24 h which corresponded well to the repair of strand breaks (Figure 1a and b).

Any significant difference from control levels in tumours exposed to 10 mg kg$^{-1}$ was not detected at any time point. This was expected since there was no induction of DNA strand breaks that could activate PARP (Figure 1), and the tumour level of NAM was not high enough to inhibit the PARP enzyme. Moreover, there was only minor induction of DNA damage assessed by alkaline elution or alteration in NAD pools determined in tumours exposed to 3aBAM, a well defined inhibitor of PARP (Table I), which may exclude the possibility that DNA damage was induced as a result of inhibition of PARP ($IC_{50}=5.4$ µM *in vivo*, Rankin *et al.*, 1989) and DNA repair.

### In vivo, *DNA damage in normal spleen tissue*

To investigate whether the induction of DNA damage was a result of the presence or absence of tumours, normal spleens originating from CBA mice without tumours, and spleens from mice carrying sarcoma A12B3 or sarcoma F tumours, were analysed by alkaline elution after exposure to single doses of NAM at 10, 100 and 1000 mg kg$^{-1}$. The A12B3 sarcoma-bearing animals had similar levels of DNA damage in their spleens compared with tumour tissues from the same mouse strain at 1.5 h, but the repair was quicker (when compared with tumour tissues (Figure 4), i.e. the half repair time was <4 h). This fact was interpreted to indicate that the induction of DNA damage by NAM is an immediate event even though the tissues are exposed to NAM for a relatively long period of time (Figure 2; Horsman *et al.*, 1993). Nevertheless, spleens from mice carrying the sarcoma F had only a minor but statistically significant increase in DNA damage at 1.5 h compared with tumour tissue (50–65% for spleen *vs* 85–100% for tumour, Figure 1b and 5). Spleen tissue from non-tumour-bearing animals did not show any significant induction of DNA damage at any dose (Figure 5), which supports the conclusion that tumour burden is essential for induction of DNA damage by NAM in spleen tissue.

### Discussion

The data reported in this study contribute two important observations to our understanding of how NAM could

**Figure 3** NAD pools in tumour tissue (**a**) A12B3 mouse sarcoma and (**b**) mouse sarcoma F as a function of time after injection (i.p.) of NAM at (□) 100 and (△) 1000 mg NAM kg$^{-1}$. NAD pools are expressed as a percentage of control ± s.e. of 3–7 exposed animals at each data point (unexposed tumours: A12B3 tumours mean: 225 ± 44 $\mu$M, $n$ = 18 and F tumours mean: 210 ± 27 $\mu$M, $n$ = 13. Indicated areas show 95% confidence interval of mean). NAD content was analysed by HPLC (see Material and methods).

**Figure 4** DNA damage and repair measured by alkaline elution in spleen tissue from tumour-bearing animals (sarcoma A12B3). Tumour-bearing CBA mice were treated with NAM at doses of (◇) no exposure, (○) 10, (□) 100 and (△) 1000 mg kg$^{-1}$, and sacrificed at indicated time points after the drug was administrated. Results are presented as means ± s.e. of 3–6 animals. *Statistically significant difference ($P$ < 0.05, Duncan test) compared with the control group.

**Figure 5** DNA damage in spleens from CBA mice (▩) without tumour, (▒) with subcutaneously transplanted sarcoma F tumours, and (▧) with subcutaneously transplanted A12B3 sarcoma tumours. The data represents DNA retained on filters as percentage of control and staples show the mean ± s.e. of 3–5 animals. *Statistically significant difference ($P$ < 0.05) compared with non-tumour-bearing animals and **statistically significant difference ($P$ < 0.05, Duncan test) compared with animals with sarcoma F tumours.

radiosensitise or induce toxic side-effects; namely (1) NAM can by itself, without co-administration of radiation, induce DNA damage and inhibit DNA repair at doses that are known to radiosensitise, and therefore, increasing tumour blood flow and overcoming tumour hypoxia is not the only radiosensitising mechanism that NAM can modulate; and (2) NAM can induce DNA damage in normal tissue of tumour-bearing animals which may in turn contribute to its toxic side-effect profile.

Our results could have been influenced by differences in tumour biology such as hypoxia or necrosis. However, we have considered this variable by always including non-treated tumour-bearing animals as a control over variations in tumour physiology for each separate experiment. As a consequence, our data has indicated that within the tumour weight range of 200–500 mg there was no measurable influence of the effects of tumour size on the NAM treatment.

The predominant radiosensitising mechanism of NAM is believed to be through increased tumour blood flow and oxygenation leading to an increase in radiation-induced DNA damage (Horsman *et al.*, 1989; Lee *et al.*, 1993; Hirst *et al.*, 1993). In this study the induction of DNA strand breaks at NAM doses of 100 mg kg$^{-1}$ and 1000 mg kg$^{-1}$ (Figures 1 and 2) could have been explained by enhanced tumour oxygenation of hypoxic cells resulting in increased production of oxygen radicals. However, this explanation cannot explain the induction of DNA damage in spleens from tumour-bearing animals where the tissue is expected to be fully oxygenated (Figure 5). Moreover, DNA damage induced by NAM in spleens from tumour-bearing animals had a repair half-time less than 4 h (Figure 4) compared with ⩾24 h in tumour tissue (Figure 1a). This point could be explained if NAM clears much faster from the spleen than from tumours, and hence there would be less of an opportunity for NAM to accumulate and inhibit DNA repair.

**Figure 6** DNA damage in spleen cell suspensions 1.5 h after injection of NAM at a dose of 1000 mg $kg^{-1}$ plotted against the average spleen weight of CBA mice; (◇) without tumours; (x) with subcutaneously transplanted sarcoma F tumours; (*) with subcutaneously transplanted sarcoma A12B3 tumours. Data points represent means ± s.e., $n \geq 3$ animals. Linear regression analysis of all the individual data points included gave a correlation coefficient of 0.885; $P<0.001$, $n=12$.

Another important consideration is that NAM had a direct dose-dependent effect on DNA damage in spleens and tumours in tumour-bearing animals (Figures 1 and 5). Whether NAM produced DNA damage by direct interaction with DNA, or by NAM triggering some other cellular metabolic event leading to DNA damage, the consequence was a fast and huge induction of DNA damage (Figures 1, 4 and 5) similar to the dose-dependent induction of DNA damage by ionising radiation alone or in combination with metoclopramide exposure shown earlier in the same experimental model (Olsson *et al.*, 1995). One plausible mechanism that could explain these results is that certain normal tissues susceptible to infiltration by phagocytes may accumulate DNA damage via activation of the respiratory burst. Endogenous oxidative stress induced by NAM in phagocytes would then be the mode of action (Shacter *et al.*, 1988; Cochrane, 1991). Support for this hypothesis has been obtained when the size of the spleens from tumour- and non-tumour-bearing animals were plotted *vs* the level of induced DNA damage in the spleens (Figure 6). The increase in weight of the spleen tissue could have been the result of an increased number of infiltrating phagocytes and leucocytes as a consequence of immunogenic tumour burden. The extent of the immunogenic response and the level of infiltration in the sarcoma A12B3 and sarcoma F tumours are currently under investigation in our laboratory. These data could be very important from a clinical perspective because they indicate that in the presence of tumour burden, NAM could cause unexpected side-effects like DNA damage to normal tissues.

The effective dose of NAM on blood flow is reported to be at concentrations from 100 mg $kg^{-1}$ and above (Chaplin *et al.*, 1990). Horsman *et al.* (1993) showed that there is no significant difference between the radiosensitising effect of NAM at doses of 100 mg $kg^{-1}$ and 1000 mg $kg^{-1}$ in a C3H mammary carcinoma, even though the difference in plasma and tumour concentrations of NAM was 5- to 10-fold. These data have suggested an alternative explanation for the radiosensitising properties of NAM; namely that NAM at tumour concentrations of 100 $\mu$M or higher inhibits the PARP enzyme ($IC_{50}=30-100$ $\mu$M) (Molinete *et al.*, 1993), which has been shown to be involved in DNA repair (Satho and Lindahl, 1994). The induced DNA damage at NAM doses of 100 and 1000 mg $kg^{-1}$ could be a result of PARP inhibition because of high tumour tissue levels of NAM (Figure 2), which in turn resulted in elevated NAD levels (Figure 3) and accumulation of DNA damage (Figure 1). Later, when NAM was cleared from tumour tissue and returned to its original, steady-state level, the DNA repair was initiated (Figures 1 and 2), and a drop in the NAD pool owing to PARP activity below control levels was expected and observed (Figure 3). Furthermore, the NAD pools in tumour tissue 1–2 h after administration of 100 mg $kg^{-1}$ or 1000 mg $kg^{-1}$ NAM was increased over controls presumably owing to PARP inhibition, and thereby, they decreased towards control levels between 24–36 h (Figure 3) when PARP was not inhibited and NAD was consumed.

In another effort to implicate PARP inhibition in the mode of action of NAM, we have used the more powerful PARP inhibitor, 3aBAM. It has been demonstrated previously that when PARP is inhibited by 3aBAM ($IC_{50}=5.4$ $\mu$M, Rankin *et al.*, 1989) instead of NAM, the NAD pools are elevated both *in vivo* and *in vitro* in normal tissues (Uchida *et al.*, 1988; Smit and Stark 1994). However, we could not show any elevation of the NAD pools in tumour tissues after exposure of tumour-bearing mice to 3aBAM at doses between 100 mg $kg^{-1}$ and 1000 mg $kg^{-1}$ (Table I). Hence these data suggest that the observed DNA-damaging effects of NAM treatment as opposed to 3aBAM treatment are more influenced by NAD metabolism supplementing NAD pools than by PARP activity which is 3–8 times higher in tumour tissue compared with normal tissues (Hirai *et al.*, 1983; Pero *et al.*, 1985; Singh, 1991). For example both 3aBAM and NAM do not 100% inhibit PARP, and so there would always be a demand for NAD. NAM treatment would support NAD synthesis whereas 3aBAM treatment could result in NAD pool depletion especially in the presence of amplified PARP activity. Lautier *et al.* (1990, 1994) showed in C3H101/2 cells, that PARP-inhibiting doses of 3aBAM (100 $\mu$M) did not inhibit NAD catabolism completely after exposure to active oxygen species even if poly(ADP-ribose) polymer production was stopped. In addition, there was no effect on the level of DNA damage or changes in the NAD pool after exposure to NAM at 10 mg $kg^{-1}$ (Figures 1 to 3, a dose not sufficient to effect PARP inhibition or blood flow). Taken together these data are consistent with the primary effects of NAM treatment being on NAD metabolism as well as on PARP inhibition when considering the possible radiosensitising mechanism of induction of DNA damage.

### Abbreviations

3aBAM, 3-aminobenzamide; DMSO, dimethyl sulphoxide; HPLC, high performance liquid chromatography; i.p., intraperitonally; NAD, nicotinamide adenine dinucleotide; NAM, nicotinamide; PARP, poly(ADP-ribose)polymerase; PCA, perchloric acid; SSB, single strand breaks; dTHd, thimidine.

### Acknowledgements

We would like to thank Dr Sally Hill, Miss Inger Andersen, Mrs Kristin Holmgren, Miss Linda Jönsson and Mrs Eva Gynnstam for their skilful technical assistance throughout these experiments. This study was supported by grants from the Swedish Cancer Society (1728-B90-01XA), the King Gustaf V Jubilee Fund, Stockholm (95:507), the Medical Faculty, University of Lund, and OxiGene Europe AB, Lund Sweden.

### References

ALISON MR, SARRAF CE, EMONS VE, HILL SA, MAGHSOUDLOO M AND MURPHY GM. (1991). Effect of alpha-difluoromethylornithine on the polyamine levels and proliferation in two transplantable tumours. *Virchows Arch. A Pathol. Anat. Histopathol.*, **419**, 223–230.

ALTHAUS FR AND RICHTER, C. (1987). *ADP-ribosylation of Proteins: Enzymology and Biological Significance*. Springer Verlag: Berlin.

BEN HUR E, UTSUMI H AND ELKIND MM. (1984). Inhibitors of poly(ADP-ribose) synthesis enhance X-ray killing of log-phase chinese hamster cells. *Radiat. Res.*, **97**, 546–555.

BERNOFSKY C. (1980). Physiologic aspects of pyridine nucleotide regulation in mammals. *Mol. Cell. Biochem.*, **33**, 135–143.

CHAPLIN DJ, TROTTER MJ, SKOV KA AND HORSMAN MR. (1990). Modification of tumour radiation response *in vivo* by the benzamide analogue pyrazinamide. *Br. J. Cancer*, **62**, 561–566.

CHAPLIN DJ, HORSMAN MR AND AOKI DS. (1991). Nicotinamide, fluosol DA and carbogen: a strategy to reoxygenate acutely and chronically hypoxic cells *in vivo*. *Br. J. Cancer*, **63**, 109–113.

COCHRANE CG. (1991). Cellular injury by oxidants. *Am. J. Med.*, **91**, 23–30.

DAI Y, YU Y AND CHEN X. (1987). The cell-cycle dependent and the DNA-damaging agent-induced changes of cellular NAD content and their significance. *Mutat. Res.*, **191**, 29–35.

DE MURCIA G, HULETSKY A AND POIRIER GG. (1988). Review: modulation of chromatin structure by poly(ADP-ribosyl)action. *Biochem. Cell Biol.*, **66**, 626–635.

DE MURCIA G, MÉNISSIER-DE MURCIA J AND SCHREIBER V. (1989). Poly(ADP-ribose)-polymerase: molecular biological aspects. *BioEssays*, **13**, 455.

ELLIOT RB AND CHASE HP. (1991). Prevention of delay of type I (insulin-dependent) diabetes mellitus in children using nicotinamide. *Diabetologica*, **35**, 362–365.

EUROPEAN SOCIETY FOR THERAPEUTIC RADIOBIOLOGY AND ONCOLOGY (ESTRO). (1994). Proceedings of the 13th Annual Meeting. 26–28 September 1994. Granada, Spain.

GEORGE AM, LUNEC J, CRAMP WA, BRENNAN S, LEWIS PD AND WISH WJD. (1986). The effects of benzamide ADP-ribosyl transferase inhibitors on cell survival and DNA strand-break repair in irradiated mammalian cells. *Int. J. Radiat. Biol.*, **49**, 783–798.

GREEN RG. (1970). Subclinical pellagra: its diagnosis and treatment. *Schizophrenia*, **2**, 70–79.

GREENBAUM GHC. (1970). An evaluation of niacinamide in the treatment of childhood schizophrenia. *Am. J. Physiatry*, **127**, 89–92.

HIRARI K, UEDA K AND HAIAISHI O. (1983). Abberation of poly(adenosine diphosphate-ribose) metabolism in human colon adenomatous polyps and cancers. *Cancer Res.*, **43**, 3441–3444.

HIRST DG, JOINER B AND HIRST VK. (1993). Blood flow modification by nicotinamide and metoclopramide in mouse tumours growing in different sites. *Br. J. Cancer*, **67**, 1–6.

HORSMAN MR. (1995). Nicotinamide and other benzamide analogs as agents for overcoming hypoxic cell radiation resistance in tumours. *Acta Oncol.*, **34**, 571–587.

HORSMAN MR, CHAPLIN DJ AND BROWN JM. (1989). Tumor radiosensitization by nicotinamide: a result of improved blood perfusion and oxygenation. *Radiat. Res.*, **181**, 139–150.

HORSMAN MR, HØYER M, HONESS DJ, DENNIS IF AND OVERGAARD J. (1993). Nicotinamide pharmacokinetics in humans and mice: a comparative assessment and the implications for radiotherapy. *Radiother. Oncol.*, **27**, 131–139.

JONSSON G, KJELLÉN E AND PERO RW. (1984*a*). Nicotinamide as a radiosensitizer of a C3H mouse mammary adenocarcinoma. *Radiother. Oncol.*, **1**, 349–353.

JONSSON G, ERIKSSON E AND PERO RW. (1984*b*). Effects of gamma radiation and hyperthermia on DNA repair synthesis and the level of NAD: cultured human mononuclear leukocytes. *Radiat. Res.*, **97**, 97–107.

KAANDERS JHAM, POP LAM, MARRES HAM, VAN DER MAAZEN RWM, VAN DER KOGEL AJ AND VAN DAAL WAJ. (1995). Radiotherapy with carbogen and nicotinamide in head and neck cancer: feasibility and toxicity. *Radiother. Oncol.*, **37**, 190–198.

KAUFMAN SH, DESNOYERS S, OTTAVIANO Y, DAVIDSON NE AND POIRIER GG. (1993). Specific proteolytic cleavage of poly(ADP-ribose)-polymerase: an early marker of chemotherapy-induced apoptosis. *Cancer Res.*, **53**, 3976–3985.

KJELLÉN E, PERO RW AND NILSSON P. (1988). Comparison of low dose nicotinamide versus benzamide, administered per os, as radiosensitizers in a C3H mammary carcinoma. *Radiother. Oncol.*, **12**, 327–331.

KJELLÉN E, JOINER MC, COLLIER JM, JOHNS H AND ROJAS A. (1991). A therapeutic benefit from combining normobaric carbogen or oxygen with nicotinamide in fractionated X-ray treatments. *Radiother. Oncol.*, **22**, 81–91.

LAUTIER D, POIRIER D, BOUDREU A, JAMALI MAA, CASTONGUAY A AND POIRIER GG. (1990). Stimulation of poly(ADP-ribose) synthesis by free radicals in C3H10T1/2 cells: relationship with NAD metabolism and DNA breakage. *Biochem. Cell Biol.*, **68**, 602–608.

LAUTIER D, HOFLACK JC, KIRKLAND JB, POIRIER D AND POIRIER GG. (1994). The role of poly(ADP-ribose) metabolism in response to active oxygen cytotoxicity. *Biochim. Biophys. Acta*, **1221**, 215–220.

LEE I, LEVITT SH AND SONG CW. (1993). Improved tumour oxygenation and radiosensitization by combination with nicotinamide and pentoxifylline. *Int. J. Radiat. Biol.*, **64**, 237–244.

MOLINETE M, VERMEULEN W, BÜRKLE A, KÜPPER JH, HOEIJMAKERS JHJ AND DE MURCIA G. (1993). Overproduction of the poly(ADP-ribose) polymerase DNA-binding domain blocks alkylation-induced DNA repair synthesis in mammalian cells. *EMBO J.*, **5**, 2109–2117.

OLSSON A, PERO RW AND OLOFSSON T. (1993). Specific binding and active transport of nicotinamide in human leukemic K562 cells. *Biochem. Pharmacol.*, **45**, 1191–1200.

OLSSON A, SHENG Y, KJELLÉN E AND PERO W. (1995). *In vivo* tumor measurement of DNA damage, DNA repair and NAD pools as indicators of radiosensitization by metoclopramide. *Carcinogenesis*, **16**, 1029–1035.

PERO RW, OLOFSSON T, GUSTAVSSON A AND KJELLÉN E. (1985). Adenosine diphosphate ribosyl transferase in marrow cells of patients with acute myeloid leukemia is related to differentiation and drug sensitivity. *Carcinogenesis*, **6**, 1055–1058.

RANKIN PW, JACOBSON EL, BENJAMIN RC, MOSS J AND JACOBSON MK. (1989). Quantitative studies of inhibitors of ADP-ribosylation *in vitro* and *in vivo*. *J. Biol. Chem.*, **264**, 4312–4317.

RAWLING JM, DRISCOLL ER, POIRIER GG AND KIRKLAND JB. (1993). Diethyl-nitrosamine administration *in vivo* increases hepatic poly(ADP-ribose) levels in rats: results of a modified technique for poly(ADP-ribose) measurement. *Carcinogenesis*, **14**, 2513–2516.

SATOH MS, POIRIER GG AND LINDAHL T. (1994). Dual function for poly(ADP-ribose) synthesis in response to DNA strand breakage. *Biochemistry*, **33**, 7099–7106.

SATHO MS AND LINDAHL T. (1994). Enzymatic repair of oxidative DNA damage. *Cancer Res.*, **54**, 1899–1901.

SHACTER E, BEECHAM EJ, COVEY JM, KOHN KW AND POTTER M. (1988). Activated neutrophils induce prolonged DNA damage in neighboring cells. *Carcinogenesis*, **9**, 2297–2304.

SINGH N. (1991). Enhanced poly ADP-ribosylation in human leukemia lymphocytes and ovarian cancers. *Cancer Lett.*, **58**, 131–135.

SMIT JS AND STARK JH. (1994). Inhibiting the repair of DNA damage induced by gamma irradiation in rat thymocytes. *Radiat. Res.*, **137**, 84–88.

VAN DER MAAZEN RWM, THIJSSEN HOM, KAANDERS JH, DE KOSTER A, KEYSER A, PRICK JA, GROTENHUIS HA, WESSELING P AND VAN DER KOGEL. (1995). Conventional radiotherapy combined with carbogen breathing and nicotinamide for malignant gliomas. *Radiother. Oncol.*, **35**, 118–122.

UCHIDA K, TAKAHASHI S, FUJIWARA K, UEDA K, NAKAE D, EMI Y, TSUTSUMI M, SHIRAIWA K, OHNISHI T AND KONISHI Y. (1988). Preventive effect of 3-amino benzamide on the reduction of NAD levels in rat liver following administration of diethylnitrosamine. *Jpn J. Cancer Res.*, **79**, 1094–1100.

ZACKHEIM HS, VASILY DB, WESTPHAL ML AND HASTINGS CW. (1981). Reactions to niacinamide. *J. Am. Acad. Dermatol.*, **4**, 736–737.